# Hemilaminectomy for the removal of the spinal tumors: An analysis of 901 patients

**DOI:** 10.3389/fneur.2022.1094073

**Published:** 2023-01-11

**Authors:** Dengyong Liao, Dan Li, Ruoran Wang, Jianguo Xu, Haifeng Chen

**Affiliations:** ^1^Department of Neurosurgery, West China Hospital, Sichuan University, Chengdu, China; ^2^Department of Physiology, School of Basic Medical Sciences, Chengdu University of Traditional Chinese Medicine, Chengdu, China

**Keywords:** hemilaminectomy, epidural, intramedullary, pain, outcome, schwannoma, meningioma

## Abstract

**Objective:**

We report our experience with the use of hemilaminectomy approach for the removal of benign intraspinal tumors.

**Method:**

A retrospective review of 1,067 patients who underwent hemilaminectomy in our hospital between 2013 and 2019 was analyzed. Baseline medical data were collected. One hundred sixteen patients were excluded due to degenerative diseases, spinal bone tumors, and malignant tumors. The remaining 901 patients (916 tumors) were enrolled. The Dennis Pain Scale (DPS) was used to assess improvement in pain before surgery and during long-term follow-up. Neurological status was assessed using the American Spinal Injury Association (ASIA) impairment scale.

**Results:**

The age of the patients was 48.7 ± 15.3 years, the duration of symptoms was 16.5 ± 32.0 months, and the tumor size was 2.6 ± 1.4 cm. Three hundred two tumors were located in the cervical region, 42 in the cervicothoracic region, 234 in the thoracic region, 57 in the thoracolumbar region, and 281 in the lumbar and lumbosacral region. Twenty-three tumors were ventrally located, 677 were dorsal or dorsolateral, 63 were intramedullary, 87 were epidural, and the rest were dumbbell-shaped. The most common pathologies were schwannomas (601, 66.7%) and meningiomas (172, 19.1%). Total excision was achieved at 97.8%. The operative time was 94.3 ± 32.6 min and the blood loss during surgery was 96.9 ± 116.5 ml. The symptom of pain improved in 87.0% of patients during long-term follow-up, neurological function improved in 68.3% and remained unchanged at 30.5%.

**Conclusion:**

The hemilaminectomy approach was a rapid and safe procedure to remove intradural and extradural tumors. This approach has offered several advantages. It could be used for the resection of most extradural or intradural extramedullary lesions, even some intramedullary tumors.

## Introduction

Today, the rationale for surgical excision of intraspinal tumors is to completely remove the tumor, maintain spinal stability and restore neurological function (1–3). Traditional laminectomies, which have been widely used in spinal surgery, are believed to provide adequate exposure to the spinal cord, nerve roots, and tumors, and thus may minimize intraoperative damage to the spinal cord (4). However, this procedure may result in persistent pain and spinal deformity after surgery due to the destruction of the posterior bony structures, supraspinal ligaments, interspinous ligaments, and paraspinal muscles bilaterally (5). Tarantino et al. (6) recently reported that about 59.6% of patients experienced severe back or wound pain within a year after using the bilateral approach.

From the recently published literature, we find that a growing number of surgeons have already preferred and advocated hemilaminectomy for the removal of spinal tumors. Previously, this approach was mainly used for small, lateral tumors (7, 8). Because of the limited and inadequate surgical corridors, the surgeons were concerned about the incomplete removal of the tumors or inadvertent damage to the spinal cord (8). Only recently has it been recognized as a valuable surgical option (9). With the advent of minimally invasive surgery (MIS), there has been an increasing emphasis on reducing the amount of bone and ligament resection during spinal surgery. While achieving the same surgical outcomes, it can reduce postoperative back pain, decrease blood loss, shorten hospital stays, and maintain spinal stability. The hemilaminectomy approach conforms to the concept of MIS, thus reducing the incidence of complications, such as infection and cerebrospinal fluid leakage (CSF) (10).

As one of the biggest neurosurgical centers in China, we first published our experience in Chinese with a series of 542 patients with intraspinal tumors who underwent surgery using hemilaminectomy in 2010 (11). At present, we describe our experience with a large series of 901 patients for whom hemilaminectomy was used for these spinal lesions, and we discuss the value of this technique and analyze possible predictors associated with better outcomes, with the aim of providing scientific and reliable evidence for the use of this procedure in clinical practice.

## Materials and methods

### Patient characteristics

The local ethics committee of our institution approved this study, and patient consent was obtained from all patients enrolled in this study. Data were reviewed retrospectively from the clinical database of our hospital for patients between 2013 and 2019. Baseline medical data, such as age, sex, duration of symptoms, preoperative neurological function, imaging data, and follow-up results were included. Intraoperative data were obtained from surgical records. Changes in pain were assessed preoperatively and during follow-up by the DPS as follows: P1: no pain; P2: occasional pain or minimal pain, no need for medication; P3: moderate pain, occasionally need for medication, but no interruption of daily activities or work; P4: moderate pain, occasionally absent from work, significant changes in daily activities; P5: constant, severe pain, chronic pain needs for the medication (9). Preoperative and long-term follow-up neurological status was assessed by the ASIA impairment scale (12). As described in the literature, the ASIA scale is classified into Grades A–E. Grades A to C on the ASIA scale were defined as a severe neurological disability. Grade D or E on the ASIA scale was defined as a mild neurological disability. All patients underwent spinal MRI preoperatively and during follow-up. The size of the tumor was defined by the largest diameter measured on the preoperative MRI. The tumors were classified as cervical, cervicothoracic, thoracic, thoracolumbar, and lumbar based on their locations, and segmented into ventral, dorsal, intramedullary, epidural, and dumb-bell tumors according to the axial location of the spinal cord. Postoperative complications were recorded, with CSF leakage and infection.

### Surgical information

The minimally invasive hemilaminectomy approach that we used has been documented in previous publications (13, 14). Before surgery, a spinal MRI revealed the tumor, and an X-ray was performed the night before surgery to mark the location of the tumor. For locating the cervical tumors, clinical palpation of the C-2 or C-7 vertebrae was an appropriate guide. Most of the patients took the prone position, and some of the patients with tumors located in the cervical spine were in the lateral position, with the side of the lesion facing up. A small midline incision was performed in the skin, the muscle was dissected from the spinal process on one side, and a Weitlaner retractor or more was used to retract the muscle to expose the lamina, preserving the lateral supraspinous and interspinous ligaments as well as the lateral attached paraspinal muscles. Hemilaminectomy was carried out using a high-speed drill (Medtronic, USA), and the soft tissue and ligamentum flavum were removed with a Kerrison rongeur to expose the dura. Tried not to remove more than 1/3 of the articular process if necessary, and the continuity between the bulk of the spinous process and the contralateral lamina should be ensured when dealing with the spinous process. For dumbbell-shaped tumors that grew into the intervertebral foramen, we often needed to remove part of the articular process. If tumors that grew beyond the midline to the contralateral side, we occasionally needed to remove the base of the spinous process. For some small tumors that grew on one side, unilateral laminectomy was usually sufficient. Then after opening the dura and arachnoid membrane, the tumor was exposed and removed microscopically either as a whole or in pieces, depending on the consistency and size of the tumor. The ventral tumor could be removed by cutting the dentate ligament, part of the articular surface, and an appropriate rotation of the operating table for visualization, allowing the tumor to be removed without manipulation of the spinal cord. Internal decompression followed by segmental resection was performed using an ultrasonic aspirator (CUSA, Integra, Inc. USA) for larger tumors. For meningiomas, the dura was partially removed or the attachment site was coagulated. For schwannoma, the affected root was normally coagulated and resected. After tumor resection, the dura was closed mainly with 6-0 proline (Ethicon, Inc.) and fibrin sealant and fat graft were applied to reinforce the closure. Somatosensory evoked potential (SSEP) and motor evoked potential (MEP) monitoring were routinely performed intraoperatively. For tumors resected in more than four laminae, we routinely placed drain tubes and removed most of them within 24–48 h after surgery in the absence of CSF leakage. In all patients with postoperative incisional CSF leakage, we routinely performed a lumbar subarachnoid drain insertion and the patients were routinely bed-rested for a few days to allow the leakage to subside. After conservative treatment, the CSF leakage was often stopped.

### Postoperative management

The pain was relieved with narcotic analgesic within 24 h of surgery, followed by nonsteroidal anti-inflammatory drugs for 3–5 days. If requested, the patients might also receive prolonged analgesic treatment. In the absence of complications, patients were often encouraged to get out of bed 3 days after surgery and be discharged home 4–7 days postoperatively. Prolonged hospital stays were usually necessary for patients who developed complications as well as those with pre-existing comorbidities.

### Follow-up

Follow-up data were collected mainly through outpatient reviews and telephone interviews. We considered 6 months to be an appropriate time for clinical evaluation of surgical outcomes, with reports indicating that neurological recovery beyond 6 months to a year was difficult (15). The patient's neurological recovery and pain were recorded during follow-up, the spinal segment where the tumor was located was examined for recurrence by MRI, and stability was assessed by X-ray. Postoperative follow-up was at a mean time of 18.4 months (6 months−4 years). Those with < 6 months of follow-up were excluded.

### Statistical analysis

All statistical analyses were performed using SPSS software (version 26.0). *T*-test, Pearson's χ^2^ test and binary logistic regression analysis were used to analyze the factors associated between the improved and unimproved groups. The statistical significance was *P* < 0.05.

### Illustrative case

**Figure 1:**
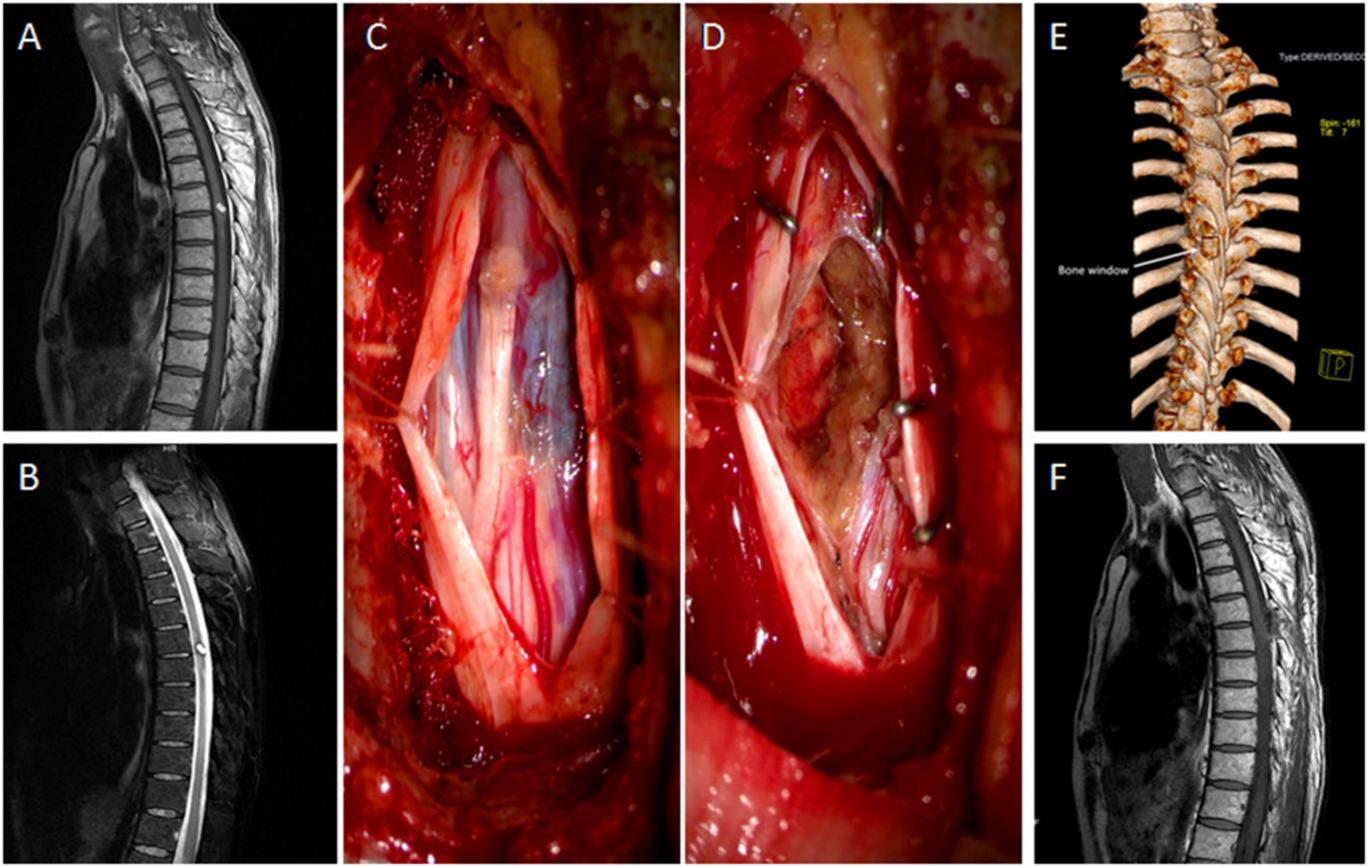
**(A, B)** The sagittal views of MRI showed mixed heterogenous signal intensity of the central core on T1-weighted image and peripheral hypointense ring of hemosiderin on T2-weighted image in the midline of the dorsal surface in the T6–7. **(C)** The tumor was exposed, with blue surface in the dorsal of the spine. **(D)** The tumor was removed. **(E)** The bone window in postoperative 3D CT scan was evident after 2 left laminae excised. **(F)** MRI taken after 6 months in sagittal view with no tumor residual or recurrence.

Sixty-three-year-old male with 2-month muscle strength class 2 in both legs. MRI showed a mixed heterogeneous signal intensity of peripheral hemosiderin rings on T2-weighted images and a central core at the midline of the dorsal surface on T1-weighted images in T6–7. A left lateral laminectomy was performed. By cutting the root of the spinous process and turning the operating table, the lesion was removed “*en bloc*” under the microscope and pathological diagnosis was cavernous hemangioma after surgery. At follow-up, the patient was able to walk freely on level surfaces, but needed support on stairs and had mild subjective numbness in both lower extremities (Figure 1).

## Results

### Patient characteristics

A total of 1,067 patients who underwent hemilaminectomy were included in the analysis, of which 49 were excluded due to degenerative disease or tumors associated with osseous destruction, as these lesions had an impact on spinal stability independent of the choice of procedure, and 117 histologically characterized malignancies with poor prognosis were also excluded. Finally, 901 patients (916 tumors) were enrolled in this subset, 452 females and 449 males, with a mean age of 48.7 ± 15.3 years. All patients underwent hemilaminectomy for resection of the tumor. No deaths due to this technique were recorded in the series. Preoperatively, 516 (57.3%) patients complained of pain, 419 (46.5%) of paresthesia, 241 (26.7%) of motor weakness, and 49 (5.4%) of sphincter dysfunction. The mean duration of symptoms was 16.5 months (9 h−252 months). Of all 916 tumors, 302 (33.0%) tumors were located in the cervical region, 42 (4.6%) in the cervicothoracic region, 234 (25.5%) in the thoracic region, 57 (6.2%) in thoracolumbar region, and 281 (30.7%) in the lumbar and lumbosacral region. According to the axial location of the tumor in the spinal cord, 87 (9.5%) tumors were epidural, 700 (76.4%) subdural extramedullary, and 63 (6.9%) intramedullary. Eight hundred and ninety-six (97.8%) tumors were completely removed, and 20 (2.2%) were subtotally resected. Sub-total resected tumors were primarily due to the lack of a clear interface between the tumor and the medulla, such as lipomas. The operative time was 94.3 ± 32.6 min, the intraoperative blood loss was ~96.9 ± 116.5 ml, and the number of resected laminae was 2.0 ± 0.8 (1–6 laminae). Histopathologic diagnoses were confirmed as schwannoma (601, 66.7%), meningioma (172, 19.1%), cavernous hemangioma (40, 4.4%), epidermoid cyst (22, 2.4%), hemangioblastoma (21, 2.3%), lipoma (8, 0.9%), and others (37, 4.1%), including teratoma, neurofibroma, ganglioneuroma, vascular malformation, and bronchogenic cyst, etc. (Table 1).

**Table 1:** General characteristics of patients.

**Characteristics**	**Patients (*n* = 901)**
**Sex**	
Female (*n*, %)	452 (50.2%)
Male (*n*, %)	449 (49.8%)
Age (years, mean ± SD)	48.7 ± 15.3
**Clinical signs**	
Pain (*n*, %)	516 (57.3%)
Sensory deficits (*n*, %)	419 (46.5%)
Motor deficits (*n*, %)	241 (26.7%)
Bowel/ urinary dysfunction (*n*, %)	49 (5.4%)
Duration of symptoms (months)	16.5 ± 32.0
**Location (*****n*** = **916, %)**	
Cervical	302 (33.0%)
Cervicothoracic	42 (4.6%)
Thoracic	234 (25.5%)
Thoracolumbar	57 (6.2%)
Lumbar and lumbosacral	281 (30.7%)
Involved segments (mean ± SD)	2.0 ± 0.8
Size (cm)	2.6 ± 1.4
**Surgery information (*****n*** = **916, %)**	
Totally removed	896 (97.8%)
Sub totally removed	20 (2.2%)
Dorsal lesions	677 (73.9%)
Ventral lesions	23 (2.5%)
Intramedullary	63 (6.9%)
Epidural	87 (9.5%)
Surgery time (min)	94.3 ± 32.6
Hemorrhage during surgery (ml)	96.9 ± 116.5
**Pathology**	
Schwannoma	601 (66.7%)
Meningioma	172 (19.1%)
Cavernous hemangioma	40 (4.4%)
Epidermoid cyst	22 (2.4%)
Hemangioblastoma	21 (2.3%)
Lipomyoma	8 (0.9%)
Other	37 (4.1%)
Hospital stay (days)	6.7 ± 4.6
Follow-up (months)	17.9 (6–48)

After surgery, patients were kept in bed for about 3 days and they were subsequently encouraged to get out of bed and walk as soon as possible. The overall average time of hospitalization was 6.7 ± 4.6 days before discharge. Postoperative complications included CSF leakage (14, 1.6%) and infection (12, 1.3%), all of whom recovered quickly after constant drainage of CSF of the lumbar cistern as well as treatment with sensitive antibiotics. During the mean follow-up time of 18.4 months (6–48 months), 719 (79.8%) patients completed long-term follow-up, 182 patients (20.2%) were lost to follow-up, and the lost to follow-up data were within acceptable limits.

### Long-term follow-up results

The pain was assessed using the DPS. Declines from high to low grades were defined as improvements, such as P5–P4, and grades that remained unchanged or increased were defined as stable or deteriorative, respectively. All 516 patients experienced pain before surgery, as follows: P2 (24, 4.7%), P3 (213, 41.3%), P4 (241, 46.7%), and P5 (38, 7.4%). During long-term follow-up, a total of 432 (83.7%) patients were assessed using the DPS, and 84 (16.3%) were lost to follow-up (Table 2). Compared with preoperative pain, patients with pain in grades P4 and P5 had significantly decreased. Specifically, during the long-term follow-up, 376 patients (87.0%) reported improvement with a decrease in pain intensity, duration, and frequency, 52 patients (12.0%) reported no change in pain, and only four patients (1%) reported aggravation of pain (Table 2).

**Table 2:** Postoperative pain improvement of the patients.

**Status**	**Dennis Pain Scale**					
	**Total**	**P1**	**P2**	**P3**	**P4**	**P5**
Preoperative	516	–	24	213	241	38
Long-term outcomes	432	134	232	64	2	–
Stable or worsened	56	–	17	37	2	–
Improved	376	–	7	135	206	28
Lost to follow-up	84	–	–	41	33	10

The neurological dysfunctions of 558 patients were assessed using the ASIA scale both preoperatively and during follow-up. The patients with preoperative neurological dysfunction were classified as follows: Grade A (2, 0.4%), Grade B (11, 2.0%), Grade C (84, 15.1%), and Grade D (461, 82.6%). Four hundred and thirty-six (78.1%) patients were assessed using the ASIA scale during long-term follow-up, and 122 (21.9%) were lost to follow-up. Follow-up time ranged from 6 to 48 months. Compared with pre-operative neurological dysfunction, outcomes in grades A, B, and C decreased obviously, indicating a significant improvement in neurological status. Specifically, in the long-term follow-up, 298 patients (68.3%) showed improvement, 133 patients (30.5%) reported no shift in symptoms, and five patients (1.2%) had deterioration (Table 3).

**Table 3:** Postoperative neurological improvement of the patients.

**Status**	**ASIA scale**					
	**Total**	**A**	**B**	**C**	**D**	**E**
Preoperative	558	2	11	84	461	0
Long-term outcomes	436	–	1	16	156	263
Worsened	5	–	–	–	5	–
Stable	133	–	–	10	123	–
Improved	298	2	8	56	232	–
Lost to follow-up	122	–	3	18	101	–

Referring to previous studies on hemilaminectomy for the removal of intraspinal tumors, the association between improved and unimproved group of pain or neurological dysfunction at follow-up was estimated using a binary logistic regression analysis. Age, sex, duration of symptoms, tumor location, axial location, total removal, tumor size, and pathology were included in the multivariate model. As shown in Table 4, there were statistically significant differences between the two groups in terms of age, duration of symptoms, and tumor location (*P* < 0.05; Table 4).

**Table 4:** Logistic regression analysis between improved and unimproved group.

**Status**	***P*-value**	**OR**	**95% confidence interval**
Age	0.035	1.015	1.001–1.028
Sex	0.430	0.850	0.568–1.273
Duration of symptoms (months)	0.000	1.043	1.033–1.053
Tumor location	0.018	0.859	0.756–0.974
Axial location	0.453	1.060	0.910–1.234
Total removal	0.345	0.381	0.051–2.831
Mean size of tumors	0.492	1.052	0.911–1.214
Pathology	0.197	1.093	0.955–1.251

A total of 719 patients underwent long-term follow-up, with some undergoing X-rays to assess spinal stability. However, imaging data for the remaining patients were not available due to reluctance to perform tests. In response to this subset of patients, we focused on cases with significant kyphosis at follow-up or the need for re-vertebral fusion surgery as a surrogate for postoperative spinal instability. In the end, we did not record any patients with spinal instability throughout the follow-up period.

## Discussion

The theoretical basis of spinal stability is the preservation of the integrity of its anatomical structure, including bony structures and the attached ligaments and muscles. The “three-column” concept of the spine, proposed by Denis in 1983 (16), laid the biomechanical foundation of spinal stability, demonstrating that preservation of the integrity of the posterior column structure is of great importance in maintaining the stability of the spine. As described by Ogden et al. (17), there is a strong correlation between the overall extent of resection of the posterior elements and the mobility of the vertebrae during axial loading.

Although traditional laminectomy or laminoplasty for the resection of intraspinal tumors has the advantage of broad exposure, it suffers from drawbacks such as extensive tissue damage, increased blood loss, and more complications (8, 18, 19). In addition, the laminectomy can lead to potential spinal instability after surgery, because the interspinous ligaments, paraspinal muscles, spinous processes and laminae, and the yellow ligaments are all destroyed bilaterally, the absence of these elements is prone to spinal instability, deformity, and epidural fibrosis after surgery (20). Although kyphoscoliosis after laminectomy is commonly asymptomatic, it is associated with long-term spinal axial pain and deterioration of spinal cord function. The cause of these progressive problems may be related to extensive surgical dissection and the paucity of muscles associated with the denervation of the paraspinal muscle complex (21–24). In particular, the children have been reported deformities of up to 88% after laminectomy, with 27%−60% of the patients requiring a second fusion procedure (25, 26). Although laminoplasty can better avoid postoperative spinal instability, it has several disadvantages including long operation time, maximized trauma, and maximal intraoperative blood loss.

Minimally invasive hemilaminectomy has become increasingly popular over the past two decades because of the low incidence of spinal instability, mild postoperative pain, and shortened hospital stays (10, 13, 26–30). As a result, hemilaminectomy has become one of the leading surgical modalities for the treatment of degenerative diseases or tumors of the spine. Because only the root of the spinous process and half of the spinal lamina on the side of the lesion were resected, the contralateral supraspinous ligaments, interspinous ligaments, paraspinal muscles, and bony structures were left intact to minimize postoperative pain, and maximize spinal stability (10, 26–28). Chiou et al. (31) reported their experience of resecting 256 spinal tumors through either hemilaminectomy or laminectomy. They compared the two approaches and concluded that the unilateral approach could be applied to epidural tumors without significant adverse effects and that it was even considered superior to the laminectomy in intradural extramedullary tumors. Sario-glu et al. (32) reported their experience with unilateral laminectomy in 40 patients and further demonstrated that, with the aid of microsurgical techniques, unilateral laminectomy could be used for all spinal tumors except those that invaded the dura extensively bilaterally. Yasargil et al. (14) and others (28, 31, 32), with their experience in microneurosurgery, recommended and pioneered hemilaminectomy for nearly any type of intradural extramedullary tumors. Some authors have presented their experience with hemilaminectomy for the resection of partial intramedullary tumors, and could also achieve favorable results (14, 31, 33). In addition, it has been reported that a median myelotomy could be performed in the surgical area *via* the hemilaminectomy approach (34), and Sun et al. (26) described a potential application of the hemilaminectomy in all lateral intradural lesions, whether located ventrally or laterally. In our retrospective analysis, 23 ventral tumors and 63 intramedullary tumors were resected by hemilaminectomy with favorable outcomes. Therefore, we generally agree with their proposals.

Compared to laminectomy, hemilaminectomy has several advantages, such as shorter surgery time, less intraoperative blood loss, shorter hospital stays, and reduced postoperative pain (9, 31). Turel et al. (13) reported in 2015 that the operative time for hemilaminectomy was 1.6 ± 0.6 h, and the patients were commonly discharged on the 2nd or 3rd day after surgery. In 2011, Naganawa et al. (27) reported that an average of 172 ± 190 g of blood loss occurred intraoperatively with hemilaminectomy, which was significantly less than that in the control group (416 ± 392 g). Recently, Pompili et al. (9) reported that late postoperative pain decreased significantly with hemilaminectomy in spinal intradural extramedullary lesions. In our study, 896 (97.8%) of 916 tumors were completely resected, the operative time was 94.3 ± 32.6 min, intraoperative blood loss was 96. 9 ± 116.5 ml and hospital stays were 6.7 ± 4.6 days. Postoperative pain and neurological symptoms improved in 376 (87.0%) and 298 (68.3%) patients, respectively. The results we reported were consistent with findings from previous studies (9, 13, 27, 31). Pain has been considered as the most common symptom after surgery. In recent years, some researchers have begun to pay attention to the problem of postoperative late pain and noted that the pain was not always associated with tumor location or histology (6, 9, 35). In these reports, 59.6% of cases developed late pain (1 year after surgery), mainly in the form of back or “wound” pain (6). In contrast, in our study, postoperative late pain was relatively nicely improved in most patients, and only two patients (0.5%) presented postoperative late pain requiring long-term medication. Severe pain of muscular and/or spinal nerve roots can prolong hospitalization, cause patient discomfort and depression, increase rehabilitation costs, and lead to medical litigation (32, 35). In an era of increasing emphasis on health economics, the benefit of an equally effective treatment that reduces hospital stays and reduces medical expenditure cannot be overemphasized. At long-term follow-up, the patients with improved or stable neurological status were observed in 431 cases (98.9%) and deteriorated in only five cases (1.1%). In this series, total resection of the tumors was not achieved in 20 (2.2%) cases because of unclear margins and close adhesion to the spinal cord. Iacoangeli et al. (36) reported 86 cases of hemilaminectomy for spinal tumor excision, in which the stability of the spine was not affected. In our study, no spinal instability was recorded during long-term follow-up, leading us to believe that hemilaminectomy was effective in maintaining postoperative spinal stability.

Several factors are thought to predict neurological outcomes in patients with spinal tumors, including age, sex, duration of symptoms, preoperative neurological function, tumor size, location, pathology, and recurrence. In the spinal meningioma prognostic evaluation scale (SPES) proposed by Frati et al. (37), the more severe the neurological status, the worse the outcomes would be. Ciappetta et al. (38) noted that the longer the duration of symptoms, the worse the prognosis, they also showed that older patients had poorer recovery of neurological symptoms than younger patients. In our study, we noted that patients with poor preoperative neurological function (ASIA 4–5) had more significant postoperative improvement, while sensory disturbances were more difficult to recover. One possible explanation is that the duration of symptoms is significantly shorter in patients who present with severe functional deficits, patients tend to seek medical attention soon after becoming unable to walk or having sphincter dysfunction, and the prognosis tends to be better after timely surgical resection of the tumor. In contrast, the willingness to seek medical attention in mild sensory disturbances is not particularly strong, resulting in a typically longer duration of symptoms. In our cohort, the age of the improved group was 47.7 ± 15.6 years, and the unimproved group was 51.4 ± 14.5 years. The results were consistent with those of Ciappetta. Therefore, we recommend early resection of symptomatic spinal tumors. Subramanian et al. (15) reported that the location of the tumor might affect spinal cord function. The cervical spinal cord was better able to tolerate tumor compression. However, the prognosis for upper thoracic tumors was generally worse than for cervical tumors. In our analysis, it was also found that the location of the tumor had an impact on the prognosis, and the difference was statistically significant.

The complications of postoperative CSF leakage and infection were reported in 14 (1.6%) and 12 patients (1.3%) of our cohort, respectively, and all patients recovered with conservative treatment. Koch-Wiewrodt et al. (39) reported that hemilaminectomy was effective in reducing the incidence of postoperative CSF leakage and infection if the removed lamina was ≤ 4 segments. We also found that in patients who developed complications of CSF leakage in our series, the number of resected laminae intraoperatively was 3.2 ± 1.5, which was higher than the average data (2.0 ± 0.8) of all patients. Therefore, if the number of resected laminae exceeds 4 intraoperatively, the risk of CSF leakage is increased. We routinely perform epidural drainage for 24–48 h, especially for the lesion located in the lumbar spine.

Regarding the downsides of hemilaminectomy, most surgeons agree that the main problems are limited tumor exposure and high surgical technique requirements. In our experience that has also been reported previously, the window of the cervical spine has the widest exposure, ~1.5–2.5 cm, and the lumbar second, ~1.5–2.0 cm, while the thoracic has the narrowest exposure, ~1.0–1.5 cm (11). Therefore, we believe that this exposure width is sufficient for most one-sided tumor resections. It is particularly crucial to note that if the window is inadequate for tumor resections, the base of the spinous process, part of the articular process, and the nearby pedicles (usually combined with resection of the articular process) can be removed for better exposure, and additional exposure can occasionally be achieved by rotating the operating table. To increase the rate of total resection and surgical outcomes, the indications for hemilaminectomy must be rigorously considered. Our experience is: (I) epidural and intradural extramedullary lateral tumors; (II) benign tumors with intramedullary laterally growing, well-circumscribed tumors such as cavernous angiomas and small hemangioblastomas. Previous studies suggested that the location of the tumor determines the choice of hemilaminectomy, and that the size of the tumor had little effect on the choice of this procedure (11, 14, 26, 32). We used to have similar views. In recent years, however, our notions have changed. Since the spinal cord can be very fragile due to compression from a slowly growing tumor, its function can easily be compromised by any incorrect spinal cord traction. Therefore, we recommend laminectomy when the spinal cord is extremely thin due to compression from a large tumor. Laminectomy is also recommended for calcified anterolateral meningiomas, bilateral epidural lesions, large lesions that are scalloped around the spinal canal, haemorragic hemangioblastoma and intramedullary malignancies such as ependymoma and astrocytoma with unclear margins. In surgery, in these cases, we usually recommend conversion from hemilaminectomy to laminectomy: the tumor is poorly exposed; the boundary of malignant tumors is unclear; significant spinal cord edema and an expected rapid recurrence of the malignancy. When we use the hemilaminectomy procedure for spinal surgery, we should also pay attention to the following points: (I) accurate localization of the lesion segment preoperatively, we performed X-ray for localization by dorsal labeling a coin in the evening before surgery; (II) if an infiltrative growth of the intramedullary tumor is seen and intraoperative frozen pathology confirms malignancy, total laminectomy should be redirected; (III) suture the dura in a waterproof manner. If there is a defect in the dura, artificial dura, biological sealants, and adipose tissue may be used for repair; (IV) if more than four laminae are removed, we routinely place a drainage tube in the epidural space for 24–48 h (11).

This study also has some limitations. First, we performed a retrospective analysis of patients who underwent a single surgical strategy, and the lack of a control group was a major limitation of this study. Second, some patients were only followed for 6 months, which was too early to assess postoperative spinal stability. As a result, additional follow-up was required to obtain additional clinical data. Third, imaging was not performed during follow-up in some patients, so there was no data for direct measurements of spinal stability. Fourth, 182 patients (20.2%) were lost to follow-up. However, we analyzed clinical data from missed follow-up patients and found little difference between follow-up and missed follow-up patients. Therefore, we implied that there was no obvious difference between the two in terms of prognosis.

## Conclusion

Unilateral hemilaminectomy has significant advantages in resecting spinal tumors, such as shorter surgery time, less intraoperative blood loss, shorter hospital stays, reduced postoperative pain, and significantly reduced spinal instability, thus avoiding the need for repeated spinal fixation surgery. We believe that it should be used as the first option in spinal lesion removal. However, due to the relatively narrow surgical window, surgical indications must be strictly controlled, and additional training in microsurgery is required for surgeons before this approach can be used.

## Data availability statement

The raw data supporting the conclusions of this article will be made available by the authors, without undue reservation.

## Author contributions

DeL collected data, analyzed it, and drafted the manuscript. DaL performed analysis and literature review. RW collected data of the articles. HC and JX provided the conception, reviewed, and edited the manuscript. All authors contributed to the article and approved the submitted version.
